# GARCHNet: Value-at-Risk Forecasting with GARCH Models Based on Neural Networks

**DOI:** 10.1007/s10614-023-10390-7

**Published:** 2023-05-22

**Authors:** Mateusz Buczynski, Marcin Chlebus

**Affiliations:** 1 Faculty of Economic Sciences, University of Warsaw, Dluga 44/50, Warsaw, Poland; 2 Interdisciplinary Doctoral School, University of Warsaw, Dobra 56/66, Warsaw, Poland

**Keywords:** Value-at-risk, GARCH, Neural networks, LSTM

## Abstract

This paper proposes a new GARCH specification that adapts the architecture of a long-term short memory neural network (LSTM). It is shown that classical GARCH models generally give good results in financial modeling, where high volatility can be observed. In particular, their high value is often praised in Value-at-Risk. However, the lack of nonlinear structure in most approaches means that conditional variance is not adequately represented in the model. On the contrary, the recent rapid development of deep learning methods is able to describe any nonlinear relationship in a clear way. We propose GARCHNet, a nonlinear approach to conditional variance that combines LSTM neural networks with maximum likelihood estimators in GARCH. The variance distributions considered in the paper are normal, t and skewed t, but the approach allows extension to other distributions. To evaluate our model, we conducted an empirical study on the logarithmic returns of the WIG 20 (Warsaw Stock Exchange Index), S&P 500 (Standard & Poor’s 500) and FTSE 100 (Financial Times Stock Exchange) indices over four different time periods from 2005 to 2021 with different levels of observed volatility. Our results confirm the validity of the solution, but we provide some directions for its further development.

## Introduction

Uncertainty in financial markets has been a main point of risk-related research for decades (Segal et al., 2015; Vorbrink, 2014). The market standard, which was established more than 30 years ago for use as a measure of risk, is Value-at-Risk (VaR) (Duffie & Pan, 1997). It is the simplest way to express potential losses over a target time horizon with a specified statistical certainty. The simplicity of VaR does not stop numerous approaches from being proposed (Engle & Manganelli, 2004; Barone-Adesi et al., 2008; Wang et al., 2010). Such scientific abundance dictates that various approaches to calculating VaR can be in use and still be considered “good.” Whether a model can be referred to as qualitatively good is a point of debate among many researchers in the field (Abad et al., 2014; Nozari et al., 2010; Ergün & Jun, 2010; Degiannakis et al., 2012; Escanciano & Olmo, 2010; Abad & Benito, 2013). Even when models have been thoroughly tested and found to be statistically valid, insufficient attention is still paid to temporal changes in financial time series characteristics, which can lead to overestimation or underestimation of risk. The best example of such situation is the financial crisis of 2008 (Degiannakis et al., 2012) or the more recent market crash caused by COVID-19 (Omari et al., 2020). Therefore the financial industry—both regulators and financial institutions—are turning to a better, probabilistic way of estimating risk based on past events that is able to quickly adjust to recent shocks (So & Philip, 2006). As of writing, the official estimate of market risk is either Value at Risk or Expected Shortfall (ES) proposed by Basel Committee. It estimates the expected value of a potential loss if such a loss on a given asset is less than VaR.

One of the most influential drivers of risk is variance, particularly its changing temporal structure or tendency to cluster (Cont, 2002). There exists a broad family of models that aim to capture such effect, the most common being Generalized Autoregressive Conditional Heteroskedasticity (GARCH) model, proposed by Bollerslev (1986). Since financial markets typically exhibit known stylized facts, a more fitting approach is to use a fat tail distribution (Aloui & Mabrouk, 2010). The introduction of distributions such as t-distributions or GEDs, which allow for modeling skewness and heavy tails, has dispelled any doubts about the validity of GARCH models (BenSaïda, 2015; Bonato, 2012). Another extension of these models, proposed by Francq and Zakoïan (2004), assumes that not only the variance exhibits temporal changes, but also the mean, but in terms of financial returns, the mean is usually insignificant in the long run (Fama, 1998).

On the other hand, financial researchers are much keen on implementing machine learning methods (Sezer et al., 2020). Deep neural networks (deep NNs) are considered a good substitute for conventional statistical methods, not only in the field of financial markets, but also in other areas of science (Mnih et al., 2013; Devlin et al., 2018; Cho et al., 2014). However, for time series data, recursive approaches such as NNs with long short-term memory (LSTM) are preferable (Goodfellow et al., 2016). In addition, NNs offer a nonlinear estimator of the likelihood function (Chen & Billings, 1992). For GARCH models, the conditional variance function usually assumes a linear or very simple nonlinear relationship between the likelihood function (and also the moments of the distribution) and the observables (Glosten et al., 1993a; Nelson & Cao, 1992).

According to Lim et al. (2019), the best approach to using machine learning in the time series domain is not to fully replace statistical and econometric approaches. Rather, they propose to combine the best of both worlds, hence the idea of this paper is to model conditional variance using NN. Several studies have already been produced on the intersection of GARCH and NN models. For example, Arnerić et al. (2014) have proposed modeling time series using the GARCH model, but with an extension to RNNs called Jordan NNs. Similar studies by Kristjanpoller and Minutolo (2015, 2016) propose an ANN-GARCH model and their results show a 25% reduction in mean absolute percentage error (MAPE). Research by Kim and Won (2018) oes a step further, incorporating an LSTM layer into the neural network, reporting a 37.2% decrease in mean absolute error (MAE). Yet another approach, proposed by Jeong and Lee (2019) considers the RNN model to determine the autoregressive moving average (ARMA) process, which drives not the conditional variance, but the conditional mean. Their results reveal that this approach leads to a reduction in MAPE of about 10%.

The aforementioned studies, however, do not specifically focus on the implementation of NNs for conditional variance alone. For example, studies by Kristjanpoller and Minutolo (2015, 2016) use GARCH estimates of variability as inputs to the NN model, while Kim and Won (2018) build NNs with covariates that are parameters of artificially generated GARCH models. Our approach leans toward estimating conditional moments of an assumed distribution using NNs, such as in Rothfuss et al. (2019). The first to propose such approach were Nikolaev et al. (2011), who investigated an approach with recursive NNs (RNN) to represent conditional variance and found that incorporating nonlinear methods (RNN-GARCH) reduces model uncertainty. Further on, Liu and So (2020) consider using the LSTM NN to model conditional variance directly through the maximum likelihood approach of the density function of the assumed distribution. They showed that this method can successfully determine both the standard deviation and variance of financial returns. Another advantage of their approach is that it can use explained artificial intelligence (XAI) methods. However, instead of using estimation, they assumed the values of additional (in addition to the first and second moments) parameters of the distribution. Another research by Nguyen et al. (2019) proposes a fairly similar approach, but to a stochastic volatility (SV) model, which is related to GARCH. In their research, they propose an SV-LSTM model that uses LSTM NN instead of using the AR(1) process to model volatility. Their results indicate that the proposed approach can give better out-of-sample estimates than standard SV models.

In this paper, we propose **GARCHNet**— a conditional specification of NN-based GARCH models with extensive use of the LSTM layer. Our incentives are based on the previously raised drawbacks of GARCH and the fact that the LSTM NN is able to adequately represent any non-linear relationships found in financial time series data. We also extend previous research in this area by proposing further distributions—we propose a GARCHNet with normal, t and skewed t distributions, and provide the necessary negative log likelihood functions for all of them, which can be used as cost functions in NN back-propagation optimization algorithms.

We also propose an empirical experiment to verify the usefulness of the GARCHNet model. The experiment consists of estimating Value-at-Risk forecasts one day ahead in a window of 250 test days (approximately one trading year) and comparing them with equivalent GARCH models. The experiment was conducted on logarithmic returns of the WIG 20 index (Warsaw Stock Exchange Index; Poland), S&P 500 (Standard and Poor’s 500; the USA) and FTSE 100 (Financial Time Stock Exchange; the UK) over four different time periods (both training and test sample) from 2005 to 2021. The experiment was written and conducted in Python and pytorch (Paszke et al., 2019).

The paper is organized as follows. In Sect. 2, we present the theoretical background of GARCHNet and the necessary background for VAR backtesting. In Sect. 3, we describe the empirical experiment with data and model descriptions. In Sect. 4, we present the results of the experiment, and in Sect. 5 we include concluding remarks and paths for extending our framework in future research.

## Methodology

### GARCH Models

GARCH model with no mean (pure GARCH process) can be specified as:

1 $$\begin{aligned} \begin{aligned} r_t&= \mu _t + \epsilon _t, \\ \epsilon _t&= \sigma _tz_t, \end{aligned} \end{aligned}$$

where $r_t$ is observed time series, $\mu _t$ is conditional mean of the process and $\sigma _t$ is the conditional standard deviation of the observed time series process. $z_t$ is an innovation process and is considered to be i.i.d with unit variance, in the most straightforward approach the assumed distribution is normal: $z_t \sim \mathcal {N}(0,1)$.

Many definitions of conditional variance have already been proposed in the VaR field: standard GARCH (Bollerslev, 1986), Exponential GARCH (EGARCH) (Nelson, 1991), Integrated GARCH (IGARCH) (Engle & Bollerslev, 1986) or Glosten-Jagannathan-Runkle GARCH (GJR-GARCH) (Glosten et al., 1993b). However, in this paper, we only utilize standard GARCH(p, q) process, which defines conditional volatility as:

2 $$\begin{aligned} \sigma _t^2 = \omega + \sum _{i=1}^{q} \beta _i \epsilon_{t-i}^2 + \sum _{i=1}^{p} \gamma _i \sigma _{t-i}^2, \end{aligned}$$

where *p* and *q* are numbers of lags of conditional variance and innovation respectively, $\pmb {\beta }$ and $\pmb {\gamma }$ are parameter vectors to be estimated. The of stationarity of the GARCH process is satisfied by the fact that $\sum _{i=1}^{q} \beta _i + \sum _{i=1}^{p} \gamma _i < 1$.

As for optimizing this process, one possible procedure is to use quasi maximum likelihood (QML). Given that the innovations are assumed to be independent, the conditional log likelihood of a vector of demeaned observed time series $\pmb {\epsilon }$ of length *T* can be defined as the sum of all log conditional densities of particular innovations $\epsilon _t$ (see Francq & Zakoïan, 2004):

3 $$\begin{aligned} \ell (\pmb {\theta }; \pmb {\epsilon }) = \sum _{t=1}^{T} \ell _t(\pmb {\theta }; \epsilon _t) = \sum _{t=1}^{T} log f(\epsilon _t|\epsilon _{t-1}, \ldots , \epsilon _1; \pmb {\theta }) = \sum _{t=1}^{T} log f(\epsilon _t; \pmb {\theta }), \end{aligned}$$

where $\pmb {\theta } = (\omega , \beta _1, \ldots , \beta _q, \gamma _1, \ldots , \gamma _p)$ is a vector of parameters, $f(\epsilon _t|\epsilon _{t-1}, \ldots , \epsilon _1; \pmb {\theta })$ is conditional density function of innovation $\epsilon _t$, however, given that the innovations are independent it will reduce to $f(\epsilon _t; \pmb {\theta })$.

Quasi maximum likelihood estimation of the parameters vector $\pmb {\theta }$ is a solution $\hat{\pmb {\theta }}$ of:

4 $$\begin{aligned} \hat{\pmb {\theta }}= & {} {\mathop {{{\,\mathrm{arg\,max}\,}}}\limits _{\theta }} \ell (\pmb {\theta }, \pmb {\epsilon }) \end{aligned}$$

5 $$\begin{aligned} \ell (\pmb {\theta }, \pmb {\epsilon })= & {} \frac{1}{T} \sum _{t=1}^T \ell _t(\pmb {\theta }, \epsilon _t) \end{aligned}$$

In the case $z_t$ is normally distributed, conditional log likelihood function for one observation is equal to:

6 $$\begin{aligned} \ell _t(\pmb {\theta }, \epsilon _t) = -\frac{1}{2} log \sigma ^2_t - \frac{1}{2} \frac{\epsilon _t^2}{\sigma ^2_t} , \end{aligned}$$

which comes down to a logarithm of a normal density function.

In the case $z_t$ is t distributed, an additional parameter is necessary to be estimated - $\eta$ - number of degrees of freedom of this distribution, with an assumption of $\eta > 2$. Therefore the parameter vector is $\pmb {\theta } = (\omega , \beta _1, \ldots , \beta _q, \gamma _1, \ldots , \gamma _p, \eta )$, and conditional log likelihood for one observation is:

7 $$\begin{aligned} \ell _t(\pmb {\theta }, \epsilon _t) = \log \Gamma \left( \frac{\eta +1}{2}\right) -\log \Gamma \left( \frac{\eta }{2}\right) -\frac{1}{2}\log (\pi \left( \eta -2\right) \sigma _t^{2}) -\frac{\eta +1}{2}\log \left( 1+\frac{\epsilon _t^{2}}{\sigma _t^{2}(\eta -2)}\right) , \end{aligned}$$

where $\Gamma (\cdot )$ is a gamma function and the log likelihood is a logarithm of density of t distribution.

In the last case, we treat $z_t$ as skewed t distributed. One more parameter is introduced -$\lambda$, responsible for the skewness of the distribution. A particular analytical implementation of the skewed t-distribution was proposed by the Hansen (1994). In this case, an additional assumption is that $-1< \lambda < 1$. Parameter vector is once again extended to $\pmb {\theta } = (\omega , \beta _1, \ldots , \beta _q, \gamma _1, \ldots , \gamma _p, \eta , \lambda )$.

8 $$\begin{aligned} \ell _t = \ln \left[ \frac{bc}{\sigma }\left( 1+\frac{1}{\eta -2} \left( \frac{a+bx/\sigma }{1+sgn(x/\sigma +a/b)\lambda }\right) ^{2}\right) ^{-\left( \eta +1\right) /2}\right] , \end{aligned}$$

where

9 $$\begin{aligned} a=4\lambda c\frac{\eta -2}{\eta -1}, \quad b^{2}=1+3\lambda ^{2}-a^{2}, \quad c=\frac{\Gamma \left( \frac{\eta +1}{2}\right) }{\sqrt{\pi \left( \eta -2\right) } \Gamma \left( \frac{\eta }{2}\right) }, \end{aligned}$$

All of the log likelihood functions are numerically obtainable. In addition, the specific form of the conditional variance does not affect the QML in the above form. It is much more influenced by the assumed distribution. This opens up the possibility of using much more complicated nonlinear forms, such as NN (Goodfellow et al., 2016).

### LSTM Neural Networks

Long Short Term Memory (LSTM) neural networks are an extension of recurrent neural networks (RNNs), proposed by Rumelhart et al. (1986). RNNs are a special type of neural networks that introduce recursion by allowing the use of sequential, autocorrelated data. The sequence (or observed time series) is accompanied by a hidden input, a kind of memory state that stores information provided with previous time steps. The next input in the sequence is predicted using this recursive hidden state:

10 $$\begin{aligned} h_t = g(W_x x_t + W_h h_{t-1} + b_h), \end{aligned}$$

where $g(\cdot )$ is an activation function (e.g., logistic sigmoid, hyperbolic tangent or Rectified Linear Unit (ReLU)), $\pmb {x} = (x_1, x_2, \ldots , x_T)$ is the sequence of observed time series of length *T*, while $\pmb {h} = (h_1, h_2, \ldots , h_T)$ represents a random vector—hidden state of the same length *T*. $W_x$ and $W_h$ are weight matrices (parameters) of the neural network, corresponding to $\pmb {x}$ and $\pmb {h}$ respectively and $b_h$ is a bias vector. Such equation assumes that the sequence can be of infinite length or at least an arbitrarily large number *T*, but due to computational obstacles (such as the problem of vanishing or exploding gradients (Pascanu et al., 2012)) the sequence length *T* is practically limited to only a few timesteps.

The problem mentioned above is practically solved by introduction of LSTM (Hochreiter & Schmidhuber, 1997). LSTMs expand the idea of hidden states by introducing gating mechanisms, which tell whether to preserve or ignore the input from the hidden state. Given that, LSTMs can “remember” or “forget” particular timesteps if necessary, building the long-term dependency parameter matrix. In detail, there are three gates: forget, input and output.

The following equations calculated iteratively build up LSTM network:

11 $$\begin{aligned} i_t= & {} g(W_{ix} x_t + W_{ih} h_{t-1} + W_{ic} c_{t-1} + b_i) \end{aligned}$$

12 $$\begin{aligned} f_t= & {} g(W_{fx} x_t + W_{fh} h_{t-1} + W_{fc} c_{t-1} + b_f), \end{aligned}$$

13 $$\begin{aligned} c_t= & {} f_t \odot c_{t-1} + i_t \odot tanh(W_{cx} x_t + W_{ch} h_{t-1} + b_c), \end{aligned}$$

14 $$\begin{aligned} o_t= & {} g(W_{ox} x_t + W_{oh} h_{t-1} + W_{oc} c_t + b_o), \end{aligned}$$

15 $$\begin{aligned} h_t= & {} o_t \odot h(c_t), \end{aligned}$$

16 $$\begin{aligned} y_t= & {} W_{yh} h_t + b_y, \end{aligned}$$

where *W* terms denote weight matrices (e.g.: $W_{ix}$ is a matrix of weights from the input gate to the input *x*), the *b* terms denote bias vectors (e.g. $b_i$ is the input gate bias vector), $g(\cdot )$ and $h(\cdot )$ denote sigmoid and hyperbolic tangent activation functions respectively here, *i*, *f* and *o* denote input, forget and output gates respectively, $c_t$ is another hidden state vector, specifically named cell activation vector (responsible for activating specific gates). The output of the neural network can be any distribution $p(\pmb {y} | \pmb {x}; \pmb {\theta })$, however most often some particular moment of this distribution is estimated directly—in our case we would like it to be conditional variance.

### GARCHNet

Our idea of a GARCH process specification is to use a neural network as an approximation of the true conditional variance specification. To optimize the NN, the likelihood functions described in Sect. 2.1 come to our aid. They are used as cost functions—in negative log likelihoods form. In GARCHNet, the GARCH specification is as follows:

17 $$\begin{aligned} l_n = W_{l_n l_{n-1}} l_{n-1} + b_{l_n} \end{aligned}$$

and

18 $$\begin{aligned} l_1 = W_{ly} y_t + b_{l_1}, \end{aligned}$$

where $y_t$ is calculated as in Eq. 16 and *n* determines the number of following fully connected layers. Given that, conditional variance is the function of the last *n* fully connected layer:

19 $$\begin{aligned} \sigma ^2_t = g(W_{Vl} l_n + b_V), \end{aligned}$$

where $g(\cdot )$ is a function with non-negative output (e.g. softplus), while $W_{Vl}$ is a matrix of weights from the last hidden layer to the output layer and $b_V$ denotes its bias. The input of such an LSTM neural network is *p* of the last observed realizations of the time series (selected earlier). Its output will be an estimate of the conditional variance. Because of the specific mechanism that drives the forgetting mechanism of LSTM layers, we do not have to worry that the sequence that is fed into the model may be too long. NNs are typically optimized using a backpropagation algorithm (Goodfellow et al., 2016), which includes calculating gradients for each neuron in the layer and then iteratively applying changes in weights based on the value of the cost function.

However, in the density functions of t and skewed t distributions, there are two additional parameters that are necessary to be estimated or assumed. In our scenario these parameters are estimated with the same NN as a function of time. Therefore degrees of freedom $\eta$ and skewness $\lambda$ are estimated as:

20 $$\begin{aligned} \eta= g(W_{El} l_n + b_E) + 2, \end{aligned}$$

21 $$\begin{aligned} \lambda= h(W_{Sl} l_n + b_S), \end{aligned}$$

where $g(\cdot )$ is a function with non-negative output (e.g. softplus), $h(\cdot )$ is a function with output in the range $(-1, 1)$, while *W* are matrices of weights from the last hidden layer to the particular output layer (degrees of freedom $\eta$ and skewness $\lambda$ respectively) and *b* vectors denote their biases. Please note that we are adding two units to the output of degrees of freedom $\eta$ to meet the assumption that $\eta > 2$. A complementary approach would imply changes in the log likelihood function.

This means that in the most advanced scenario, for skewed t distribution, there are three last hidden layers (one for conditional variance $\sigma _t^2$, one for degrees of freedom $\eta$ and one for skewness $\lambda$), each resulting in one different output neuron.

Originally, conditional variance’s parameters ($\omega$, $\beta$ and $\gamma$) should be non-negative (Bollerslev, 1986), which together with non-negativity of random variables ($\sigma _t^2$ and $z_t^2$) suffices for the conditional variance to be non-negative as well. In the case of neural network, such assumption could lead to the worsening of the accuracy of estimated solution (Chorowski & Zurada, 2014). Instead of using such limitation, we have proposed to use softplus function (or any other that outputs non-negative values and is easily differentiable). Softplus function is defined as:

22 $$\begin{aligned} \text {Softplus}(x) = log(1 + exp(x)). \end{aligned}$$

In the case of skewness we have proposed to use hyperbolic tangent function so that the output meets the assumption that $-1< \lambda < 1$. Hyperbolic tangent function is defined as:

23 $$\begin{aligned} \tanh (x) = \frac{\exp (x) - \exp (-x)}{\exp (x) + \exp (-x)}. \end{aligned}$$

### Value-at-Risk

Value-at-Risk (VaR) defines the worst possible loss with a given probability $\alpha$, assuming normal market conditions for a specific time period *t* (Philippe, 2006). In other words, VaR is a quantile of the distribution of the observed financial time series. In our case, these are log returns of the price quotations of the respective stock index.

24 $$\begin{aligned} P(r_{t}<VaR_{\alpha } (t)|\Omega _{t-1} )=\alpha , \end{aligned}$$

where $r_t$ is the realization of the observed financial time series and $\Omega _{t-1}$ is an information set given at the time $t-1$.

When parametric models are employed, such as GARCH, VaR is calculated as an $\alpha$ quantile of the assumed innovation distribution—$F^{-1}(\alpha )$ (*F* is inverse cumulative distribution function of the assumed innovation distribution), weighted by the estimate of the conditional standard deviation $\sigma _t$, plus an estimate of conditional mean $\mu _t$ (Angelidis et al., 2004):

25 $$\begin{aligned} VaR_{\alpha } = \mu _t + \sigma _tF^{-1}(\alpha ). \end{aligned}$$

#### Quality of VaR Forecasts

The primary tool for assessing the quality of the VaR forecast is the number of cases in which the VaR forecast was lower (in absolute terms) than the realization of the observed time series—excess count or proportion of failures (Chlebus, 2017):

26 $$\begin{aligned} \hat{\alpha } = \frac{1}{N}\sum _{t=1}^N I_{VaR_\alpha >r_t}, \end{aligned}$$

where *N* is the number of testing instances and $\sum _{t=1}^N I_{VaR_\alpha >r_t}$ is the $\text {number of exceedances}= n$.

Statistically, this number comes from a binomial distribution (assuming the exceptions are IID). The Basel Committee strictly regulates what values constitute a “safe zone” or require a look at the model. Specifically, the name of such a test is the Traffic Light Test (Costanzino & Curran, 2018). In the case of VaR at 2.5% significance level and 250 testing instances the ’safe’ (green) zone ends with 10 exceptions (95% cumulative probability) and yellow (warning zone) ends with 16 exceptions (99.99% cumulative probability).

The unconditional coverage (UC) test by Kupiec (1995) builds up on the idea that the overall number of exceptions should follow the binomial distribution. To test that a likelihood ratio test is proposed:

27 $$\begin{aligned} LR_{UC} = -2ln\left( \frac{(1-\alpha )^{N-n}\alpha ^n}{(1-\hat{\alpha })^{N-n}\hat{\alpha }^n}\right) . \end{aligned}$$

There is also a conditional coverage (CC) test by Christoffersen (1998). In addition to the unconditional coverage, Christoffersen test measures the likelihood of unusually frequent VaR exceptions—an effect of exceptions clustering.

The conditional coverage test consists of both unconditional coverage and independence tests: $LR_{cc} = LR_{uc} + LR_{ind}$. The independence test $LR_{ind}$ verifies whether the exceptions follow a first-order Markov chain.

Even more restrictive is dynamic quantile (DQ) test by Engle and Manganelli (2004). They define another random variable $Hit_t = I_t - \alpha$. The null hypothesis of this test is that the expected value of the $Hit_t$ explained with the information available at $t-1$ is zero. To test that, they implement a linear regression model:

28 $$\begin{aligned} Hit_t = \delta + \sum _{k=1}^K \beta _k X_{t-k} + \epsilon _t, \end{aligned}$$

where matrix *X* might include both lags of *Hit*, *r* or *VaR*. DQ test statistic is then:

29 $$\begin{aligned} DQ = \frac{Hit'X(X'X)^{-1}X'Hit}{\alpha (1-\alpha )} \end{aligned}$$

Another interesting dimension of model comparison are loss functions (LF). Their value determines the loss if the model fails. There are two parties who are usually interested in these values—the regulator and the companies themselves. Both weigh certain business aspects differently. From the regulator’s point of view, the most important aspect is the value lost on the occurrence of a VaR exception, while from the company’s point of view, the opportunity cost of holding excess reserves.

To compare the models we have chosen following loss functions, from the proposed by Abad et al. (2015):

- Lopez quadratic LF (LLF):
  30 $$\begin{aligned} LLF_t = \left\{ \begin{array}{ll} 1 + (VaR_t - r_t)^2 &{} \text {if}\, r_t<VaR_t,\\ 0 &{} \text {otherwise}; \end{array}\right. \end{aligned}$$
- Caporin regulator’s LF (CRLF):
  31 $$\begin{aligned} CLF_t = \left\{ \begin{array}{ll} |1 - |r_t/VaR_t|| &{} \text {if}\, r_t<VaR_t,\\ 0 &{} \text {otherwise}; \end{array}\right. \end{aligned}$$
- Caporin firm’s LF (CFLF):
  32 $$\begin{aligned} CFLF_t = |1 - |r_t/VaR_t|| \text { for all } r_t; \end{aligned}$$
- Abad, Benito, Lopez’s LF (ABLLF):
  33 $$\begin{aligned} ABLLF_t = \left\{ \begin{array}{ll} (VaR_t - r_t)^2 &{} \text {if}\, r_t<VaR_t,\\ \beta (r_t - VaR_t) &{} \text {otherwise}, \end{array}\right. \end{aligned}$$
  where $\beta$ is a parameter that represents a cost of capital, originally an interest rate.

Gneiting (2011) also suggests to specify a scoring function for single-valued point forecasts, such as the ones that we generate in this paper. Specifically, for $\alpha$-quantile forecasts he proposes to use a generalized piecewise linear (GPL) scoring function, in a form of:

34 $$\begin{aligned} S_{\alpha , b, t} = \left\{ \begin{array}{ll} (1(x_t \ge y_t) - \alpha ) \frac{1}{|b|}(x_t^b-y_t^b) &{} \text {if}\, b \in \mathbb {R} \setminus \{0\},\\ (1(x_t \ge y_t) - \alpha ) log \frac{x_t}{y_t}&{} \text {if b = 0}, \end{array}\right. \end{aligned}$$

where *x* is a vector of predictions and *y* is a vector of realized rates of return. In case $b = 1$, we end up with asymmetric piecewise linear scoring function, which we propose to use in this paper. The overall result for a model is a sum for all the test cases.

## Data and Model Specifications

### Data

GARCHNet’s performance was measured empirically by backtesting on the log returns of price quotations of WIG20 (Warsaw Stock Exchange; Poland), S&P 500 (New York Stock Exchange, the USA) and FTSE 100 (London Stock Exchange, the UK). Therefore our observed time series is:

35 $$\begin{aligned} r_t = log p_t - log p_{t-1}. \end{aligned}$$

Such data is openly available, e.g.: from Stooq (2021). As a reference, we also estimated the corresponding GARCH models on the same data samples.

We subjectively selected four different time periods consisting of 1250 observations each (1000 was our training window length and we covered 250 test samples). The start dates of specific periods are as follows: (i) 2005-01-01 (testing on year 2009), (ii) 2007-01-01 (testing on year 2011), (iii) 2013-01-01 (testing on year 2017), (iv) 2016-01-01 (testing on year 2020). In our opinion these periods provide a full view of possible volatility levels between training window and predicted sample- training and testing samples both show low volatility (sample starting in 2013 respectively) or the volatility is different for training and testing samples (low volatility training samples starting in 2005 and 2016); and high volatility training sample starting in 2007). Such a spectrum allows us to test the model in varying market conditions.

### Models

We compared the proposed GARCHNet specifications with the corresponding standard GARCH models. To do this, we also had to select the number of observations *p*, which is the sequence for the LSTM model. Because of the similarity to the original meaning of *p* in the GARCH model (the number of lagged conditional variances in its model), we controlled both of these parameters with *p*. The test included $p \in {5, 10, 20, 100}$. In addition, we have reported the results for GARCH models with $p \in {1,2}$ assuming that a comparison with our results should also provide information on how the new models behave in relation to standard approach. We set the $\alpha$ significance level for VaR estimates at 2.5%. We also set the random seed equal to 1.

We used a rolling-window estimation approach (Zanin & Marra, 2012). For each forecast sample, we prepared a new model with new randomly initialized weights and trained it using the last 1000 observations. We have also tested a hypothesis that frequent updates of the model might not necessarily improve its quality, while only increasing the time overhead in training. We tested a framework where the model was fully reset (random weights fully initialized) less often than with each timestep forecast. The model might be refitted with fresh data, between resets to include new information. In the most extreme approach we assumed that it is only trained fully once (on the first 1000 observations) and then we have increased the frequency of training up to 500 updates (update very other training sample). Such approach has been proven faulty in results comparison, due to large jumps in volatility estimates. The results are not reported here.

**Fig. 1:**
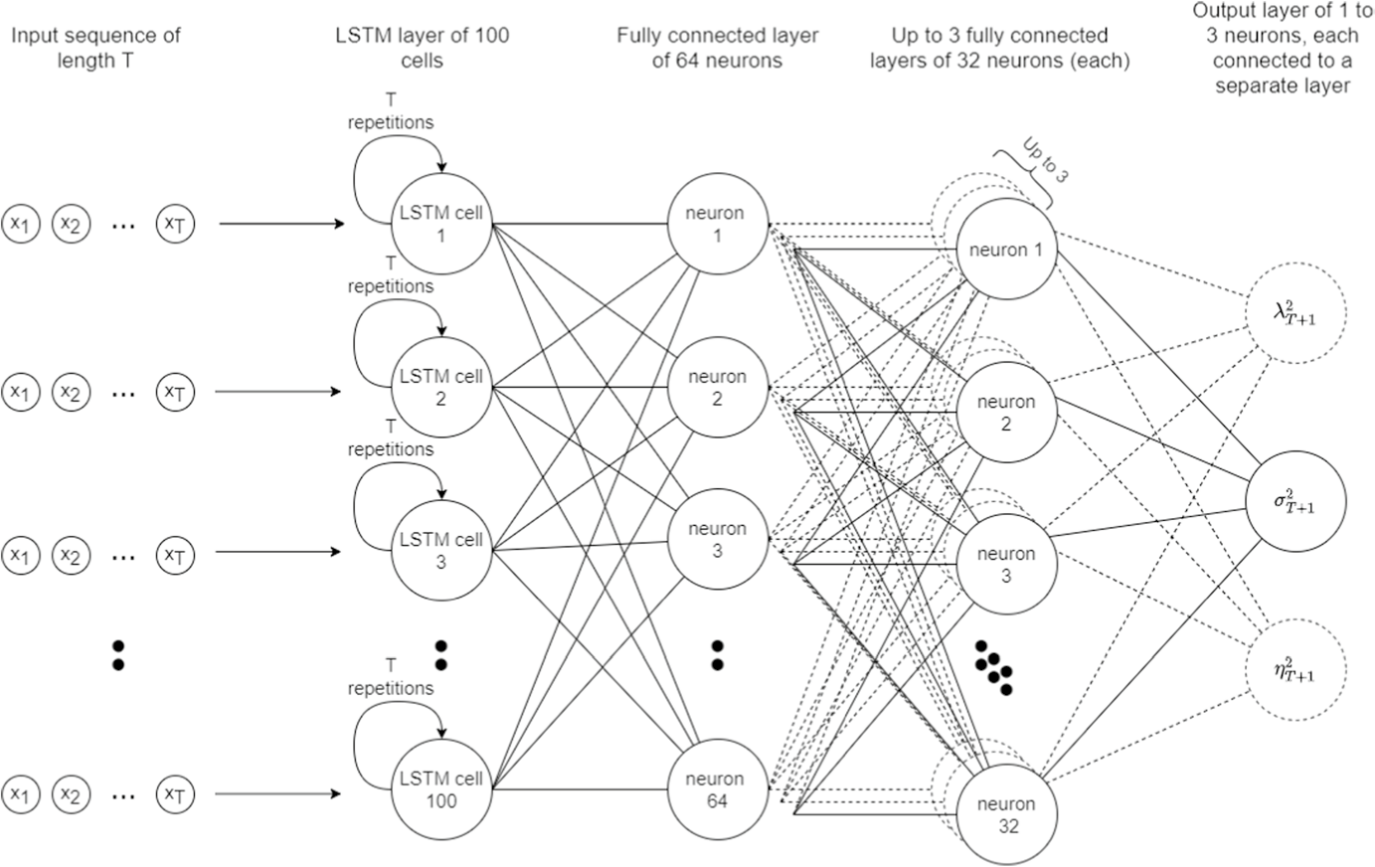
The diagram with the architecture of GARCHNet model

For the neural network determining the conditional variance, we used a rather small architecture (see Fig. 1): one LSTM layer with 100 neurons (fed by a sequence of length *p*), followed by three ($n = 3$) fully connected layers with 64, 32 and 1 neuron(s), respectively. For t and skew t distributions, there were two (and three, respectively) output layers corresponding to the number of parameters being optimized. Parameter optimization was performed using the Adam optimizer with a learning rate of 3e-4 and a batch size of 512. Due to the rolling-window method, it was difficult to choose an automatic threshold for the number of epochs to avoid overfitting, so each model was trained for 300 epochs.

## Results

**Fig. 2̄:**
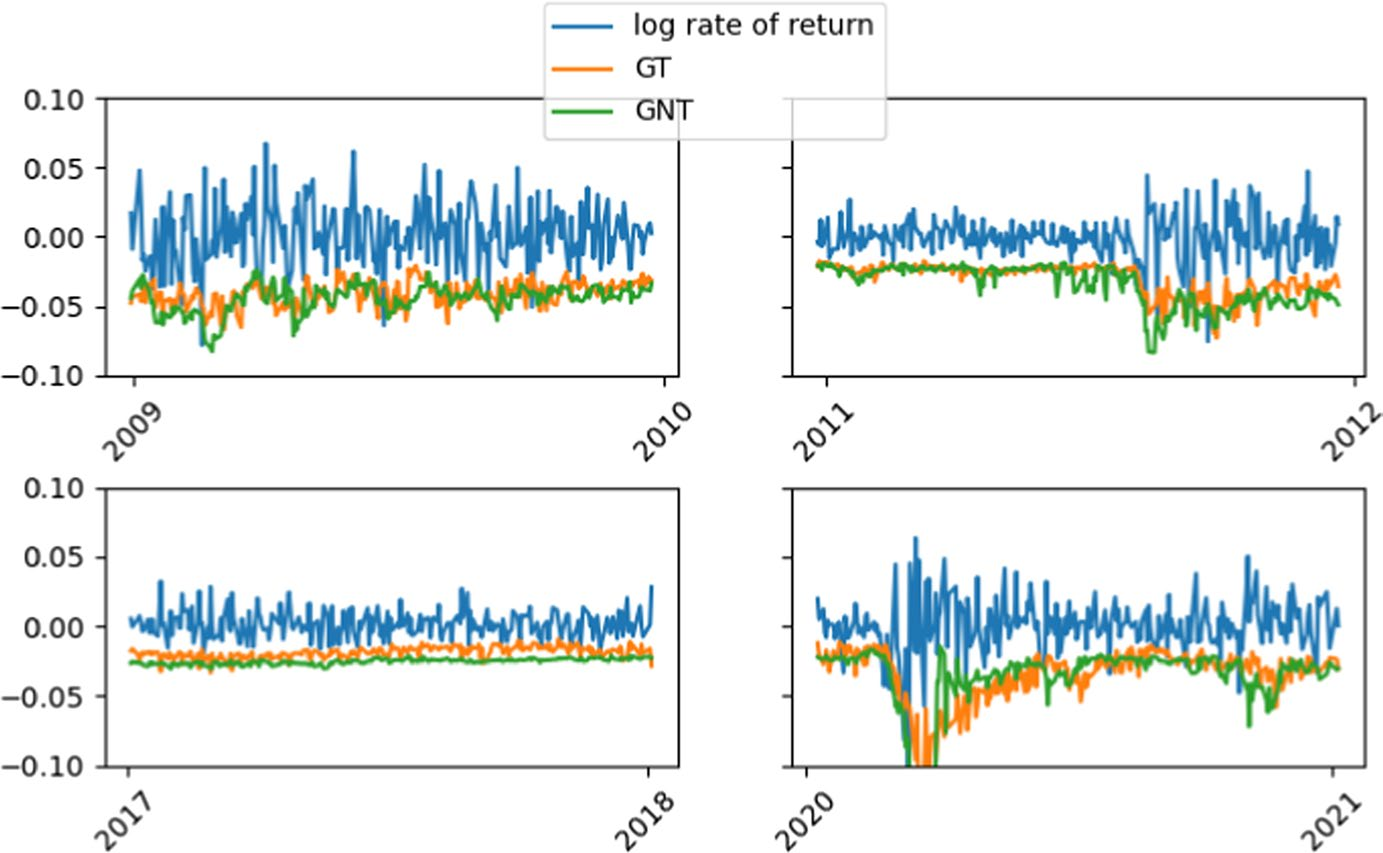
Exemplary comparison for the GARCH and GARCHNet with t distribution for $p=20$ for WIG 20. *Note*: logarithmic rate of return (in blue); GT (in orange)—GARCH with t distributed innovations; GNT (in green)—GARCHNet with t distributed innovations. (Color figure online)

The results of the experiment are satisfactory. Figure 2 shows the relationship between GARCH and GARCHnet predictions with innovations with a t distribution. It can be seen that the GARCHNet predictions do not deviate from the rate of return, even more—for some intervals GARCHNet confirms the presence of volatility shocks much faster. It can also be noted that the GARCHNet model tends to estimate a higher VaR than GARCH, except for the most recent period, where the relationship is reversed.

In the Tables 1, 2 and 3 we have presented the results of the statistical tests and the number of exceptions for each index tested. The results are mostly the same for all indexes, but the biggest difference is seen for the WIG 20. There is no GARCH model that outperforms its GARCHNet counterpart across all periods tested and for all *p* sequence lengths tested in terms of number of exceptions. However, GARCHNet with a t-distribution appears to have the largest excess. In the case of the WIG 20, for only two cases was the number of exceptions higher than for GARCH with a t distribution, for the S&P 500 it was five cases and for the FTSE 100 nine cases. It should be noted that most of these exceedances occurred in the last two periods. For the other models, the overhead is much smaller and sometimes negative. However, we believe that the predictive power of such a model could be improved with a better neural architecture.

GARCHNet with a skewed t-distribution is worse by a small margin, which is not consistent with its GARCH counterpart—GARCH with a skewed t-distribution is the best model compared to other members of its family. This may indicate that the distribution parameter estimation approach is inefficient for Adam’s optimizer, or that the approach we used should be reconsidered. For example, the parameter estimation should be changed to an estimation for the entire training sample, rather than based on a fairly small sample (of length *p*) of time series in the prediction phase. GARCHNet with a normal distribution tends to be worse than its standard counterpart, but in the last two periods this relationship is much smaller. We assume that this is due to the worse predictive power of the GARCH model in turbulent periods.

We have also prepared results for GARCH models with $p \in {1, 2}$. We note that for each GARCHNet model there is a *p* that will provide results better than the standard GARCH approach. This is most apparent for periods with a large discrepancy between the level of variability in the training and test samples. GARCHnet models have typically from 5 to 1 exceedances fewer than GARCH models with $p \in {1, 2}$.

**Table 1:** Results of GARCH and GARCHNet models regarding *p* values of considered statistical tests for WIG20

p		Period I (2009)					Period II (2011)					Period III (2017)					Period IV (2020)				
	Model	H	UC	CC	DQ	GPL	H	UC	CC	DQ	GPL	H	UC	CC	DQ	GPL	H	UC	CC	DQ	GPL
1	GN	**7.00**	**0.76**	**0.10**	**0.38**	**0.32**	**5.00**	**0.61**	0.01	**0.79**	**0.23**	**1.00**	0.01	**0.62**	0.03	**0.14**	33.00	0.00	0.00	0.00	0.55
	GT	10.00	**0.16**	0.00	**0.06**	0.37	6.00	**0.93**	0.04	**0.30**	0.26	2.00	0.05	**0.36**	**0.13**	0.14	29.00	0.00	0.00	0.00	**0.52**
	GS	9.00	**0.29**	0.00	**0.06**	0.41	6.00	**0.93**	**0.05**	**0.30**	0.27	2.00	0.05	**0.36**	**0.13**	0.14	**27.00**	0.00	0.00	0.00	0.53
2	GN	9.00	**0.29**	0.00	**0.06**	**0.35**	9.00	**0.29**	0.00	**0.35**	**0.28**	**1.00**	0.01	**0.62**	0.03	**0.13**	27.00	0.00	0.00	0.00	0.55
	GT	11.00	**0.08**	0.00	0.05	0.37	**8.00**	**0.49**	0.00	**0.39**	0.29	6.00	**0.93**	0.00	**0.30**	0.16	23.00	0.00	0.00	0.00	0.50
	GS	**8.00**	**0.49**	0.00	0.05	0.37	**8.00**	**0.49**	0.00	**0.39**	0.30	7.00	**0.76**	0.00	0.03	0.17	**21.00**	0.00	0.00	0.00	**0.49**
5	GN	10.00	**0.16**	0.00	**0.26**	0.39	9.00	**0.29**	**0.25**	**0.35**	0.27	1.00	0.01	**0.62**	0.03	**0.13**	18.00	0.00	0.00	0.00	0.48
	GT	10.00	**0.16**	0.00	**0.06**	0.39	9.00	**0.29**	**0.26**	**0.35**	0.27	1.00	0.01	**0.61**	0.03	0.13	16.00	0.00	0.00	0.00	0.44
	GS	9.00	**0.29**	0.00	**0.35**	0.39	10.00	**0.16**	0.00	**0.06**	0.27	1.00	0.01	**0.61**	0.03	0.13	15.00	0.00	0.00	0.01	**0.42**
	GNN	13.00	0.02	0.00	0.02	0.35	4.00	**0.33**	**0.09**	**0.08**	**0.26**	**0.00**	–	–	–	0.13	18.00	0.00	0.00	0.00	0.48
	GNT	**5.00**	**0.61**	**0.24**	**0.18**	0.36	**1.00**	0.01	**0.60**	0.03	0.30	**0.00**	–	–	–	0.17	15.00	0.00	0.00	0.00	0.46
	GNS	6.00	**0.93**	**0.99**	**0.86**	**0.33**	**1.00**	0.01	**0.62**	0.03	0.29	**0.00**	–	–	–	0.16	**14.00**	0.01	0.00	0.00	0.46
10	GN	7.00	**0.76**	0.00	**0.78**	0.36	11.00	**0.08**	0.00	0.05	**0.26**	1.00	0.01	**0.62**	0.03	**0.13**	**13.00**	0.02	0.00	0.00	0.53
	GT	7.00	**0.76**	0.00	**0.78**	0.37	11.00	**0.08**	0.00	0.05	0.26	1.00	0.01	**0.62**	0.03	0.13	**13.00**	0.02	0.00	0.00	0.49
	GS	6.00	**0.93**	**0.13**	**0.86**	0.37	10.00	**0.16**	0.00	**0.06**	0.27	1.00	0.01	**0.62**	0.03	0.13	**13.00**	0.02	0.00	0.00	0.50
	GNN	9.00	**0.29**	**0.11**	**0.35**	0.33	9.00	**0.29**	**0.31**	**0.35**	0.29	2.00	0.05	**0.69**	**0.13**	0.13	18.00	0.00	0.00	0.00	0.46
	GNT	**5.00**	**0.61**	**0.26**	**0.18**	0.33	**4.00**	**0.33**	**0.92**	**0.59**	0.26	**0.00**	–	–	–	0.17	17.00	0.00	0.00	0.00	**0.43**
	GNS	6.00	**0.93**	**0.99**	**0.86**	**0.32**	5.00	**0.61**	**0.26**	**0.18**	0.27	**0.00**	–	–	–	0.16	17.00	0.00	0.00	0.00	0.46
20	GN	8.00	**0.49**	**0.17**	**0.39**	0.37	8.00	**0.49**	**0.19**	**0.39**	0.27	1.00	0.01	**0.62**	0.03	**0.13**	**13.00**	0.02	0.00	0.00	0.51
	GT	7.00	**0.76**	**0.18**	**0.38**	0.37	8.00	**0.49**	**0.24**	**0.39**	0.28	1.00	0.01	**0.61**	0.03	0.13	15.00	0.00	0.00	0.00	0.48
	GS	8.00	**0.49**	0.00	**0.39**	0.39	8.00	**0.49**	**0.24**	**0.39**	0.28	1.00	0.01	**0.61**	0.03	0.13	15.00	0.00	0.00	0.00	0.47
	GNN	10.00	**0.16**	0.01	**0.06**	0.34	9.00	**0.29**	**0.33**	**0.35**	**0.26**	3.00	**0.15**	**0.63**	**0.34**	0.14	**13.00**	0.02	0.00	**0.05**	0.43
	GNT	6.00	**0.93**	**0.32**	**0.30**	0.35	5.00	**0.61**	**0.97**	**0.79**	0.26	**0.00**	–	–	–	0.17	14.00	0.01	0.00	0.01	**0.42**
	GNS	**3.00**	**0.15**	**0.93**	**0.34**	**0.32**	**4.00**	**0.33**	**0.09**	**0.08**	0.28	2.00	0.05	**0.75**	**0.13**	0.15	16.00	0.00	0.00	0.00	0.51
100	GN	4.00	**0.33**	**0.98**	**0.59**	0.36	17.00	0.00	0.00	0.00	0.32	4.00	**0.33**	**0.08**	**0.59**	**0.12**	**14.00**	0.01	0.00	0.00	0.54
	GT	3.00	**0.15**	**0.94**	**0.34**	0.36	17.00	0.00	0.00	0.00	0.32	2.00	0.05	**0.82**	**0.13**	0.12	**14.00**	0.01	0.00	0.00	0.54
	GS	**2.00**	0.05	**0.80**	**0.13**	0.36	15.00	0.00	0.00	0.00	0.33	3.00	**0.15**	0.01	**0.34**	0.13	**14.00**	0.01	0.00	0.00	0.54
	GNN	11.00	**0.08**	**0.08**	**0.17**	0.34	5.00	**0.61**	**0.25**	**0.18**	**0.28**	1.00	0.01	**0.62**	0.03	0.12	15.00	0.00	0.00	0.00	0.43
	GNT	4.00	**0.33**	**0.04**	**0.08**	0.36	**1.00**	0.01	**0.61**	0.03	0.35	**0.00**	–	–	–	0.16	**14.00**	0.01	0.00	0.01	**0.41**
	GNS	6.00	**0.93**	**0.98**	**0.86**	**0.32**	5.00	**0.61**	**0.28**	**0.79**	0.30	3.00	**0.15**	**0.77**	**0.34**	0.15	15.00	0.00	0.00	0.00	0.46

GN—GARCH with normally distributed innovations (d.i.); GT—GARCH with t d.i.; GST—GARCH with skewed t d.i.; GNN—GARCHNet with normally d.i.; GNT—GARCHNet with t d.i.; GNS—GARCHNet with skewed t d.i.; H—number of hits within test period; UC—unconditional coverage test; CC—conditional coverage test; DQ—dynamic quantile test; GPL—generalized piecewise linear scoring function

All statistical tests that were failed to be rejected at the 5% significance level are in bold. Best Hit and GPL value are also in bold

**Table 2̄:** Results of GARCH and GARCHNet models regarding *p* values of considered statistical tests for S&P 500

p		Period I (2009)					Period II (2011)					Period III (2017)					Period IV (2020)				
	Model	H	UC	CC	DQ	GPL	H	UC	CC	DQ	GPL	H	UC	CC	DQ	GPL	H	UC	CC	DQ	GPL
1	GN	24.00	0.00	0.00	0.00	0.34	7.00	**0.76**	0.00	**0.78**	0.27	3.00	**0.15**	0.00	**0.34**	**0.08**	**21.00**	0.00	0.00	0.00	**0.32**
	GT	23.00	0.00	0.00	0.00	0.34	7.00	**0.76**	0.00	**0.78**	0.26	3.00	**0.15**	0.01	**0.34**	0.08	24.00	0.00	0.00	0.00	0.34
	GS	**21.00**	0.00	0.00	0.00	**0.32**	**5.00**	**0.61**	0.00	**0.79**	**0.23**	**1.00**	0.01	**0.34**	0.03	0.08	25.00	0.00	0.00	0.00	0.34
2	GN	22.00	0.00	0.00	0.00	0.31	8.00	**0.49**	0.00	**0.60**	0.26	2.00	0.05	0.02	**0.13**	**0.07**	20.00	0.00	0.00	0.00	0.31
	GT	20.00	0.00	0.00	0.00	**0.31**	8.00	**0.49**	0.01	**0.60**	0.25	**1.00**	0.01	**0.24**	0.03	0.08	**17.00**	0.00	0.00	0.00	**0.28**
	GS	**19.00**	0.00	0.00	0.00	0.32	**7.00**	**0.76**	0.00	**0.78**	**0.25**	**1.00**	0.01	**0.50**	0.03	0.09	**17.00**	0.00	0.00	0.00	0.31
5	GN	18.00	0.00	0.00	0.00	0.34	12.00	0.04	0.00	**0.10**	0.25	3.00	**0.15**	**0.14**	**0.34**	**0.08**	13.00	0.02	0.00	0.03	0.29
	GT	18.00	0.00	0.00	0.00	0.34	10.00	**0.16**	0.00	**0.26**	0.25	3.00	**0.15**	**0.38**	**0.34**	0.08	13.00	0.02	0.00	**0.05**	0.27
	GS	17.00	0.00	0.00	0.00	0.32	9.00	**0.29**	0.00	**0.35**	0.24	**1.00**	0.01	**0.59**	0.03	0.09	**5.00**	**0.61**	**0.99**	**0.79**	**0.21**
	GNN	10.00	**0.16**	0.02	**0.24**	**0.29**	9.00	**0.29**	**0.35**	**0.35**	**0.24**	4.00	**0.33**	**0.93**	**0.59**	0.09	21.00	0.00	0.00	0.00	0.45
	GNT	**1.00**	0.01	**0.58**	0.03	0.36	**2.00**	0.05	0.00	**0.13**	0.29	2.00	0.05	**0.77**	**0.13**	0.11	7.00	**0.76**	**0.10**	**0.38**	0.40
	GNS	6.00	**0.93**	**0.11**	**0.30**	0.30	**2.00**	0.05	**0.82**	**0.13**	0.25	3.00	**0.15**	**0.84**	**0.34**	0.11	14.00	0.01	0.00	0.01	0.46
10	GN	14.00	0.01	0.01	0.01	0.32	9.00	**0.29**	0.00	**0.41**	0.25	3.00	**0.15**	**0.57**	**0.34**	**0.08**	12.00	0.04	0.00	**0.06**	0.28
	GT	14.00	0.01	0.01	0.01	0.32	11.00	**0.08**	0.00	**0.13**	0.25	3.00	**0.15**	**0.59**	**0.34**	0.08	**3.00**	**0.15**	**0.94**	**0.34**	0.22
	GS	13.00	0.02	0.02	0.03	0.31	7.00	**0.76**	0.00	**0.78**	**0.24**	**1.00**	0.01	**0.60**	0.03	0.09	**3.00**	**0.15**	**0.94**	**0.34**	**0.22**
	GNN	9.00	**0.29**	**0.15**	**0.41**	**0.29**	13.00	0.02	0.00	0.02	0.25	5.00	**0.61**	**1.00**	**0.79**	0.09	17.00	0.00	0.00	0.00	0.46
	GNT	**1.00**	0.01	**0.57**	0.03	0.31	**3.00**	**0.15**	0.01	**0.34**	0.27	3.00	**0.15**	**0.92**	**0.34**	0.10	7.00	**0.76**	**0.10**	**0.38**	0.40
	GNS	6.00	**0.93**	**0.13**	**0.86**	0.29	**3.00**	**0.15**	0.01	**0.34**	0.26	5.00	**0.61**	**0.69**	**0.79**	0.11	15.00	0.00	0.00	0.01	0.48
20	GN	11.00	**0.08**	**0.20**	**0.13**	0.30	11.00	**0.08**	0.00	**0.17**	0.27	6.00	**0.93**	**0.87**	**0.86**	0.09	6.00	**0.93**	**0.42**	**0.86**	0.24
	GT	12.00	0.04	**0.07**	**0.06**	0.30	10.00	**0.16**	0.00	**0.26**	0.27	8.00	**0.49**	**0.83**	**0.60**	0.09	**3.00**	**0.15**	**0.94**	**0.34**	0.26
	GS	10.00	**0.16**	**0.17**	**0.24**	0.30	7.00	**0.76**	0.00	**0.78**	**0.25**	6.00	**0.93**	**0.81**	**0.86**	**0.09**	**3.00**	**0.15**	**0.94**	**0.34**	**0.24**
	GNN	11.00	**0.08**	**0.08**	**0.13**	**0.29**	13.00	0.02	0.00	0.02	0.26	5.00	**0.61**	**1.00**	**0.79**	0.09	15.00	0.00	0.00	0.00	0.42
	GNT	**3.00**	**0.15**	**0.89**	**0.34**	0.33	**2.00**	0.05	0.00	**0.13**	0.28	**1.00**	0.01	**0.61**	0.03	0.11	9.00	**0.29**	**0.24**	**0.35**	0.39
	GNS	4.00	**0.33**	**0.97**	**0.59**	0.29	8.00	**0.49**	**0.06**	**0.39**	0.28	4.00	**0.33**	**0.96**	**0.59**	0.10	14.00	0.01	0.00	0.00	0.51
100	GN	7.00	**0.76**	**0.18**	**0.78**	0.32	14.00	0.01	0.00	0.01	0.31	9.00	**0.29**	**0.24**	**0.41**	0.09	10.00	**0.16**	0.00	**0.26**	**0.26**
	GT	7.00	**0.76**	**0.19**	**0.78**	0.32	14.00	0.01	0.00	0.01	0.31	8.00	**0.49**	**0.31**	**0.60**	0.09	13.00	0.02	0.00	**0.05**	0.28
	GS	7.00	**0.76**	**0.21**	**0.78**	0.33	13.00	0.02	0.00	0.03	0.30	8.00	**0.49**	**0.36**	**0.60**	0.09	19.00	0.00	0.00	0.00	0.29
	GNN	13.00	0.02	0.01	0.03	**0.28**	12.00	0.04	0.00	**0.10**	**0.25**	5.00	**0.61**	**1.00**	**0.79**	**0.09**	23.00	0.00	0.00	0.00	0.50
	GNT	**0.00**	–	–	–	0.38	**2.00**	0.05	0.00	**0.13**	0.30	**1.00**	0.01	**0.62**	0.03	0.11	**8.00**	**0.49**	**0.16**	**0.39**	0.43
	GNS	6.00	**0.93**	**0.54**	**0.86**	0.31	4.00	**0.33**	**0.07**	**0.59**	0.29	3.00	**0.15**	**0.90**	**0.34**	0.10	17.00	0.00	0.00	0.00	0.51

GN—GARCH with normally distributed innovations (d.i.); GT—GARCH with t d.i.; GST—GARCH with skewed t d.i.; GNN—GARCHNet with normally d.i.; GNT—GARCHNet with t d.i.; GNS—GARCHNet with skewed t d.i.; H—number of hits within test period; UC—unconditional coverage test; CC—conditional coverage test; DQ—dynamic quantile test; GPL—generalized piecewise linear scoring function

All statistical tests that were failed to be rejected at the 5% significance level are in bold. Best Hit and GPL value are also in bold

**Table 3:** Results of GARCH and GARCHNet models regarding *p* values of considered statistical tests for FTSE 100

p		Period I (2009)					Period II (2011)					Period III (2017)					Period IV (2020)				
	Model	H	UC	CC	DQ	GPL	H	UC	CC	DQ	GPL	H	UC	CC	DQ	GPL	H	UC	CC	DQ	GPL
1	GN	11.00	**0.07**	0.02	**0.15**	0.22	**3.00**	**0.15**	**0.36**	**0.34**	**0.17**	4.00	**0.34**	**0.45**	**0.59**	0.09	22.00	0.00	0.00	0.00	0.25
	GT	11.00	**0.07**	0.03	**0.15**	0.23	**3.00**	**0.15**	**0.39**	**0.34**	0.17	**2.00**	0.05	**0.78**	**0.14**	0.09	14.00	0.01	0.00	0.02	0.23
	GS	**7.00**	**0.71**	**0.10**	**0.76**	**0.22**	**3.00**	**0.15**	**0.35**	**0.34**	0.18	**2.00**	0.05	**0.18**	**0.14**	**0.08**	**11.00**	**0.08**	**0.06**	**0.17**	**0.22**
2	GN	12.00	0.03	0.00	0.03	0.21	4.00	**0.33**	**0.82**	**0.59**	**0.18**	**3.00**	**0.15**	**0.23**	**0.34**	**0.09**	21.00	0.00	0.00	0.00	0.26
	GT	11.00	**0.07**	0.00	0.04	0.21	**3.00**	**0.15**	**0.63**	**0.34**	0.18	**3.00**	**0.15**	**0.50**	**0.34**	0.09	10.00	**0.16**	0.00	**0.24**	**0.23**
	GS	**6.00**	**0.98**	0.03	**0.86**	**0.18**	**3.00**	**0.15**	**0.60**	**0.34**	0.19	**3.00**	**0.15**	**0.56**	**0.34**	0.09	**8.00**	**0.49**	0.00	**0.60**	0.24
5	GN	17.00	0.00	0.00	0.00	0.25	5.00	**0.61**	**0.22**	**0.18**	0.19	**3.00**	**0.15**	**0.20**	**0.34**	**0.08**	16.00	0.00	0.00	0.00	0.22
	GT	17.00	0.00	0.00	0.00	0.25	5.00	**0.61**	**0.22**	**0.18**	**0.19**	**3.00**	**0.15**	**0.68**	**0.34**	0.09	12.00	0.04	0.01	**0.10**	**0.22**
	GS	10.00	**0.14**	**0.10**	**0.24**	**0.24**	3.00	**0.15**	**0.73**	**0.34**	0.19	**3.00**	**0.15**	**0.61**	**0.34**	0.09	**7.00**	**0.76**	**0.07**	**0.38**	0.23
	GNN	13.00	0.02	0.04	**0.05**	0.27	9.00	**0.29**	0.01	**0.06**	0.24	7.00	**0.76**	**0.15**	**0.78**	0.11	21.00	0.00	0.00	0.00	0.43
	GNT	**0.00**	–	–	–	0.30	**2.00**	0.05	**0.79**	**0.13**	0.23	**3.00**	**0.15**	**0.68**	**0.34**	0.12	14.00	0.01	0.00	0.01	0.40
	GNS	6.00	**0.93**	**0.93**	**0.86**	0.24	3.00	**0.15**	**0.94**	**0.34**	0.24	**3.00**	**0.15**	**0.82**	**0.34**	0.12	13.00	0.02	0.00	**0.05**	0.41
10	GN	17.00	0.00	0.00	0.00	0.24	6.00	**0.93**	**0.98**	**0.86**	**0.18**	**3.00**	**0.15**	**0.76**	**0.34**	0.09	7.00	**0.76**	**0.55**	**0.78**	**0.21**
	GT	16.00	0.00	0.00	0.00	0.24	4.00	**0.33**	**0.91**	**0.59**	0.18	4.00	**0.34**	**0.78**	**0.59**	**0.09**	4.00	**0.33**	**0.09**	**0.59**	0.21
	GS	7.00	**0.71**	**0.49**	**0.76**	**0.22**	**3.00**	**0.15**	**0.73**	**0.34**	0.19	4.00	**0.34**	**0.90**	**0.59**	0.09	**2.00**	0.05	**0.77**	**0.13**	0.22
	GNN	10.00	**0.16**	**0.44**	**0.24**	0.25	10.00	**0.16**	0.01	**0.06**	0.22	6.00	**0.93**	**0.22**	**0.86**	0.11	17.00	0.00	0.00	0.00	0.38
	GNT	**2.00**	0.05	**0.81**	**0.13**	0.29	4.00	**0.33**	**0.93**	**0.59**	0.22	4.00	**0.33**	**0.94**	**0.59**	0.11	13.00	0.02	0.00	0.02	0.35
	GNS	5.00	**0.61**	**0.91**	**0.79**	0.26	**3.00**	**0.15**	**0.92**	**0.34**	0.23	5.00	**0.61**	**0.36**	**0.79**	0.13	12.00	0.04	0.00	0.03	0.34
20	GN	8.00	**0.45**	**0.77**	**0.57**	0.22	5.00	**0.61**	**0.20**	**0.18**	**0.18**	3.00	**0.15**	**0.66**	**0.34**	**0.08**	7.00	**0.76**	**0.96**	**0.78**	**0.22**
	GT	8.00	**0.45**	**0.88**	**0.57**	**0.21**	5.00	**0.61**	**0.20**	**0.18**	0.18	4.00	**0.34**	**0.95**	**0.59**	0.09	6.00	**0.93**	**0.99**	**0.86**	0.22
	GS	**4.00**	**0.36**	**0.99**	**0.62**	0.22	**3.00**	**0.15**	0.01	0.02	0.18	4.00	**0.34**	**0.98**	**0.59**	0.09	**4.00**	**0.33**	**0.98**	**0.59**	0.23
	GNN	12.00	0.04	0.04	**0.06**	0.26	10.00	**0.16**	0.00	0.01	0.21	8.00	**0.49**	**0.07**	**0.60**	0.11	19.00	0.00	0.00	0.00	0.39
	GNT	7.00	**0.76**	**0.89**	**0.78**	0.27	4.00	**0.33**	**0.99**	**0.59**	0.21	**2.00**	**0.05**	**0.78**	**0.13**	0.11	12.00	0.04	0.01	**0.10**	0.35
	GNS	9.00	**0.29**	**0.07**	**0.41**	0.26	4.00	**0.33**	**0.98**	**0.59**	0.22	4.00	**0.33**	**0.60**	**0.59**	0.13	15.00	0.00	0.00	0.01	0.38
100	GN	4.00	**0.36**	**0.99**	**0.62**	**0.25**	11.00	**0.08**	0.02	0.05	0.21	15.00	0.00	0.00	0.01	**0.10**	7.00	**0.76**	**0.14**	**0.78**	0.22
	GT	4.00	**0.36**	**0.99**	**0.62**	0.25	11.00	**0.08**	0.02	0.05	0.21	14.00	0.01	0.00	0.02	0.10	8.00	**0.49**	**0.12**	**0.60**	0.23
	GS	**2.00**	**0.05**	**0.84**	**0.15**	0.25	8.00	**0.49**	**0.60**	**0.39**	0.21	12.00	0.04	0.01	**0.10**	0.10	**4.00**	**0.33**	**0.97**	**0.59**	**0.22**
	GNN	15.00	0.00	0.00	0.00	0.28	7.00	**0.76**	**0.56**	**0.38**	**0.21**	8.00	**0.49**	0.03	**0.60**	0.10	24.00	0.00	0.00	0.00	0.43
	GNT	5.00	**0.61**	**0.96**	**0.79**	0.28	3.00	**0.15**	**0.86**	**0.34**	0.24	**2.00**	**0.05**	**0.81**	**0.13**	0.12	13.00	0.02	0.00	0.00	0.40
	GNS	6.00	**0.93**	**0.89**	**0.86**	0.26	**2.00**	0.05	**0.81**	**0.13**	0.23	3.00	**0.15**	**0.75**	**0.34**	0.13	12.00	0.04	0.00	0.01	0.41

GN—GARCH with normally distributed innovations (d.i.); GT—GARCH with t d.i.; GST—GARCH with skewed t d.i.; GNN—GARCHNet with normally d.i.; GNT—GARCHNet with t d.i.; GNS—GARCHNet with skewed t d.i.; H—number of hits within test period; UC—unconditional coverage test; CC—conditional coverage test; DQ—dynamic quantile test; GPL—generalized piecewise linear scoring function

All statistical tests that were failed to be rejected at the 5% significance level are in bold. Best Hit and GPL value are also in bold

**Table 4:** Results of GARCH and GARCHNet models regarding the values of cost functions for WIG 20

p		Period I (2009)				Period II (2011)				Period III (2017)				Period IV (2020)			
	Model	LLF	CRLF	CFLF	ABLLF	LLF	CRLF	CFLF	ABLLF	LLF	CRLF	CFLF	ABLLF	LLF	CRLF	CFLF	ABLLF
1	GN	**7.00**	**1.34**	**149.35**	**0.11**	**5.00**	**0.76**	**171.17**	**0.08**	**1.00**	**0.06**	**165.27**	**0.05**	33.02	28.33	186.14	**0.07**
	GT	10.00	2.37	152.50	0.11	6.00	0.92	175.43	0.09	2.00	0.94	167.80	0.05	29.01	**21.64**	**182.63**	0.07
	GS	9.00	1.98	164.05	0.13	6.00	1.11	175.40	0.09	2.00	0.98	168.68	0.05	**27.01**	27.54	187.65	0.07
2	GN	9.00	2.40	151.26	0.11	9.00	2.46	**171.50**	0.08	**1.00**	**0.00**	**164.26**	0.05	27.02	23.99	183.62	**0.07**
	GT	11.00	3.14	**150.53**	**0.11**	**8.00**	**2.45**	173.58	0.08	6.00	6.76	171.11	0.05	23.01	**14.50**	**168.64**	0.08
	GS	**8.00**	**1.66**	158.44	0.12	8.00	2.66	173.46	0.08	7.00	8.19	173.16	0.05	**21.01**	16.12	173.97	0.08
5	GN	10.00	4.36	150.33	0.11	9.00	2.37	**170.55**	**0.08**	1.00	0.08	**161.26**	**0.05**	18.01	11.81	163.84	**0.08**
	GT	10.00	4.30	150.16	0.10	9.00	2.36	170.80	0.08	1.00	0.11	161.31	0.05	16.01	8.10	157.90	0.09
	GS	9.00	3.29	154.67	0.11	10.00	2.46	170.61	0.08	1.00	0.09	161.64	0.05	15.01	7.71	155.69	0.08
	GNN	13.00	3.07	**145.98**	**0.10**	4.00	2.20	171.00	0.08	**0.00**	**0.00**	163.41	0.05	18.01	10.33	**155.58**	0.08
	GNT	**5.00**	1.27	161.73	0.12	**1.00**	**0.30**	192.77	0.11	**0.00**	**0.00**	180.42	0.07	15.01	**7.37**	164.25	0.09
	GNS	6.00	**0.99**	156.43	0.12	1.00	0.89	185.09	0.10	**0.00**	**0.00**	173.43	0.06	**14.01**	8.43	162.00	0.09
10	GN	7.00	2.80	150.07	**0.11**	11.00	2.02	168.25	0.08	1.00	0.06	**160.39**	**0.05**	13.02	13.43	170.30	0.10
	GT	7.00	2.89	151.39	0.11	11.00	2.08	168.80	0.08	1.00	0.10	160.93	0.05	**13.01**	10.68	168.74	0.10
	GS	6.00	2.22	157.48	0.12	10.00	2.32	169.18	0.08	1.00	0.09	161.92	0.05	13.01	11.11	168.57	0.10
	GNN	9.00	1.83	**148.53**	0.11	9.00	3.53	**165.76**	**0.08**	2.00	0.17	162.25	0.05	18.01	9.65	**156.53**	**0.08**
	GNT	**5.00**	0.89	158.37	0.12	**4.00**	0.85	176.82	0.09	**0.00**	**0.00**	179.22	0.07	17.01	**6.29**	161.69	0.09
	GNS	6.00	**0.60**	158.83	0.12	5.00	**0.68**	179.52	0.10	**0.00**	**0.00**	173.25	0.06	17.01	7.83	160.13	0.09
20	GN	8.00	3.26	151.99	0.11	8.00	2.61	166.98	0.08	1.00	0.14	**155.89**	**0.05**	13.01	12.52	168.87	0.10
	GT	7.00	3.13	153.64	0.11	8.00	2.88	167.53	0.08	1.00	0.17	156.87	0.05	15.01	9.31	166.77	0.10
	GS	8.00	2.66	159.02	0.12	8.00	2.91	167.65	0.08	1.00	0.16	158.53	0.05	15.01	9.55	165.46	0.10
	GNN	10.00	2.41	**148.58**	**0.10**	9.00	2.44	**160.81**	**0.07**	3.00	0.58	160.02	0.05	**13.01**	7.57	**159.94**	**0.09**
	GNT	6.00	1.49	158.92	0.12	5.00	1.23	174.39	0.09	**0.00**	**0.00**	177.80	0.07	14.01	**5.92**	167.01	0.10
	GNS	**3.00**	**0.80**	158.26	0.12	**4.00**	**0.86**	180.93	0.10	2.00	0.08	170.60	0.06	16.01	9.59	166.54	0.10
100	GN	4.00	1.18	159.74	0.12	17.00	7.76	155.69	**0.07**	4.00	0.24	**147.64**	**0.05**	14.02	14.14	172.01	0.11
	GT	3.00	1.16	160.12	0.13	17.00	7.74	156.00	0.07	2.00	0.34	148.40	0.05	14.02	14.14	172.50	0.11
	GS	**2.00**	**0.46**	167.10	0.14	15.00	8.18	**155.54**	0.07	3.00	0.35	151.60	0.05	14.02	14.75	170.15	0.10
	GNN	11.00	2.49	**148.48**	**0.10**	5.00	2.09	174.93	0.09	1.00	0.11	157.15	0.05	15.01	7.64	**158.51**	**0.09**
	GNT	4.00	0.93	165.29	0.13	**1.00**	**0.26**	201.54	0.14	**0.00**	**0.00**	173.89	0.06	**14.01**	**5.17**	163.89	0.09
	GNS	6.00	1.10	154.74	0.11	5.00	0.86	188.20	0.11	3.00	0.30	168.87	0.06	15.01	8.11	161.97	0.09

GN—GARCH with normally distributed innovations (d.i.); GT—GARCH with t d.i.; GST—GARCH with skewed t d.i.; GNN—GARCHNet with normally d.i.; GNT—GARCHNet with t d.i.; GNS—GARCHNet with skewed t d.i.

Minimum cost values for each period and p pairs are in bold

**Table 5:** Results of GARCH and GARCHNet models regarding the values of cost functions for S&P 500

p		Period I (2009)				Period II (2011)				Period III (2017)				Period IV (2020)			
	Model	LLF	CRLF	CFLF	ABLLF	LLF	CRLF	CFLF	ABLLF	LLF	CRLF	CFLF	ABLLF	LLF	CRLF	CFLF	ABLLF
1	GN	24.00	9.42	**140.49**	**0.06**	7.00	2.65	170.49	0.08	3.00	0.24	**185.72**	**0.03**	**21.00**	**22.97**	**131.91**	**0.05**
	GT	23.00	8.79	141.04	0.06	7.00	2.39	170.40	0.08	3.00	0.24	186.62	0.03	24.00	66.08	193.32	0.06
	GS	**21.00**	**7.53**	141.55	0.06	**5.00**	**1.32**	**168.64**	**0.07**	**1.00**	**0.01**	193.19	0.03	25.00	60.19	189.41	0.06
2	GN	22.00	7.70	141.03	**0.06**	8.00	3.26	167.41	0.07	2.00	**0.03**	**185.52**	**0.03**	20.00	**19.94**	**124.71**	**0.05**
	GT	20.00	**7.37**	**140.72**	0.06	8.00	3.10	**165.56**	**0.07**	**1.00**	0.07	186.39	0.03	**17.00**	21.70	151.62	0.06
	GS	**19.00**	8.27	144.61	0.06	**7.00**	**2.56**	168.71	0.07	1.00	0.13	193.45	0.03	17.00	36.73	166.88	0.06
5	GN	18.00	7.60	**150.63**	**0.07**	12.00	3.64	**163.74**	**0.07**	3.00	0.49	**183.38**	**0.03**	13.00	15.05	**131.98**	**0.06**
	GT	18.00	7.46	151.61	0.07	10.00	3.27	164.50	0.07	3.00	0.60	184.56	0.03	13.00	23.86	172.72	0.07
	GS	17.00	5.89	152.17	0.07	9.00	2.44	167.89	0.07	**1.00**	**0.26**	192.56	0.03	**5.00**	**1.44**	132.39	0.07
	GNN	10.00	3.51	160.34	0.09	9.00	2.17	164.09	0.07	4.00	1.32	184.83	0.03	21.01	14.04	151.87	0.08
	GNT	**1.00**	**0.30**	193.62	0.14	**2.00**	**0.22**	195.86	0.11	2.00	0.48	200.83	0.04	7.00	3.06	180.58	0.11
	GNS	6.00	2.89	170.12	0.10	2.00	0.57	181.74	0.09	3.00	1.16	197.31	0.04	14.01	9.60	160.44	0.09
10	GN	14.00	5.09	**155.29**	**0.08**	9.00	3.70	**162.83**	**0.07**	3.00	0.77	**179.15**	**0.03**	12.00	8.50	**137.29**	**0.07**
	GT	14.00	5.10	156.32	0.08	11.00	3.56	163.27	0.07	3.00	0.75	181.30	0.03	**3.00**	**0.94**	147.45	0.08
	GS	13.00	4.29	157.63	0.08	7.00	2.59	167.02	0.07	**1.00**	**0.31**	190.61	0.03	3.00	1.13	144.74	0.08
	GNN	9.00	3.52	158.75	0.09	13.00	3.49	163.41	0.07	5.00	2.14	181.57	0.03	17.01	15.61	151.13	0.08
	GNT	**1.00**	**0.14**	185.61	0.12	3.00	**1.09**	182.48	0.09	3.00	1.13	195.19	0.03	7.00	3.79	176.25	0.11
	GNS	6.00	1.57	172.76	0.10	**3.00**	1.18	181.36	0.09	5.00	2.15	194.80	0.04	15.01	10.22	164.94	0.10
20	GN	11.00	2.87	162.14	0.09	11.00	5.24	**162.34**	**0.07**	6.00	3.49	**170.27**	**0.02**	6.00	1.30	**152.11**	0.08
	GT	12.00	2.80	162.82	0.09	10.00	4.85	162.53	0.07	8.00	3.26	170.96	0.02	3.00	1.27	159.82	0.09
	GS	10.00	2.24	166.33	0.10	7.00	3.02	165.73	0.07	6.00	1.79	179.00	0.03	**3.00**	**0.63**	156.57	0.09
	GNN	11.00	2.85	**157.96**	**0.09**	13.00	4.01	163.35	0.07	5.00	1.76	183.39	0.03	15.01	13.23	152.31	**0.08**
	GNT	**3.00**	**1.10**	184.23	0.12	**2.00**	**1.02**	185.34	0.10	**1.00**	**0.32**	201.21	0.04	9.00	4.58	168.50	0.10
	GNS	4.00	1.22	172.16	0.10	8.00	2.48	182.00	0.09	4.00	1.10	196.60	0.04	14.01	13.76	174.42	0.10
100	GN	7.00	3.20	170.06	0.11	14.00	10.82	**159.78**	**0.06**	9.00	4.91	**164.51**	**0.02**	10.00	4.70	145.32	0.08
	GT	7.00	2.99	170.84	0.11	14.00	10.83	160.00	0.06	8.00	4.74	165.36	0.02	13.00	**2.50**	142.64	0.08
	GS	7.00	2.85	174.40	0.11	13.00	9.37	163.95	0.07	8.00	2.88	172.42	0.03	19.00	4.26	**140.20**	**0.08**
	GNN	13.00	4.46	**150.00**	**0.08**	12.00	2.78	166.65	0.07	5.00	1.69	181.49	0.03	23.01	20.16	149.77	0.08
	GNT	**0.00**	**0.00**	195.64	0.15	**2.00**	**0.17**	196.28	0.12	**1.00**	**0.19**	203.37	0.04	**8.00**	4.74	177.72	0.12
	GNS	6.00	1.98	170.62	0.10	4.00	1.12	189.40	0.10	3.00	0.73	197.44	0.04	17.01	13.49	171.97	0.09

GN—GARCH with normally distributed innovations (d.i.); GT—GARCH with t d.i.; GST—GARCH with skewed t d.i.; GNN—GARCHNet with normally d.i.; GNT—GARCHNet with t d.i.; GNS—GARCHNet with skewed t d.i.

Minimum cost values for each period and p pairs are in bold

**Table 6:** Results of GARCH and GARCHNet models regarding the values of cost functions for FTSE 100

p		Period I (2009)				Period II (2011)				Period III (2017)				Period IV (2020)			
	Model	LLF	CRLF	CFLF	ABLLF	LLF	CRLF	CFLF	ABLLF	LLF	CRLF	CFLF	ABLLF	LLF	CRLF	CFLF	ABLLF
1	GN	11.00	3.02	139.09	0.06	3.00	0.24	**160.73**	**0.06**	4.00	1.10	**167.47**	**0.03**	22.00	8.83	**139.87**	**0.05**
	GT	11.00	3.38	**138.56**	**0.06**	3.00	0.22	161.97	0.07	2.00	1.09	169.64	0.03	14.00	5.66	143.37	0.06
	GS	**7.00**	**1.96**	144.41	0.06	**3.00**	**0.12**	166.15	0.07	**2.00**	**0.41**	170.83	0.03	**11.00**	**3.58**	146.93	0.07
2	GN	12.00	2.27	136.50	0.06	4.00	0.56	**157.79**	**0.06**	3.00	1.02	**166.50**	**0.03**	21.00	9.51	**139.66**	**0.06**
	GT	11.00	2.32	**135.57**	**0.06**	3.00	0.53	159.78	0.07	3.00	0.92	169.16	0.03	10.00	5.17	141.45	0.06
	GS	**6.00**	**0.54**	143.02	0.06	**3.00**	**0.41**	164.11	0.07	**3.00**	**0.74**	171.06	0.03	**8.00**	**4.20**	148.95	0.07
5	GN	17.00	4.25	**137.41**	0.06	5.00	1.03	**155.91**	**0.06**	3.00	0.88	**165.83**	**0.03**	16.00	4.84	**136.58**	**0.06**
	GT	17.00	4.13	137.42	**0.06**	5.00	0.91	157.17	0.06	3.00	1.06	168.82	0.03	12.00	3.24	142.86	0.07
	GS	10.00	3.18	141.72	0.06	3.00	0.65	162.31	0.07	**3.00**	**0.70**	170.32	0.03	**7.00**	**2.33**	151.39	0.08
	GNN	13.00	4.45	159.11	0.07	9.00	3.32	163.92	0.07	7.00	2.40	175.37	0.03	21.01	11.16	153.83	0.08
	GNT	**0.00**	**0.00**	189.75	0.12	**2.00**	**0.25**	182.15	0.09	3.00	0.72	190.99	0.04	14.01	6.25	169.67	0.10
	GNS	6.00	0.75	170.62	0.09	3.00	0.67	182.84	0.09	3.00	0.90	191.40	0.04	13.01	8.67	162.36	0.08
10	GN	17.00	3.40	**138.21**	0.06	6.00	0.91	**155.45**	**0.06**	**3.00**	1.11	**166.79**	**0.03**	7.00	2.08	**144.59**	**0.07**
	GT	16.00	3.44	138.39	**0.06**	4.00	0.86	156.53	0.06	4.00	0.82	167.43	0.03	4.00	1.16	152.55	0.08
	GS	7.00	1.98	143.67	0.07	3.00	0.61	161.91	0.07	4.00	**0.74**	169.15	0.03	**2.00**	**0.72**	161.21	0.08
	GNN	10.00	2.86	156.25	0.08	10.00	2.97	161.26	0.07	6.00	2.74	172.87	0.03	17.01	7.85	153.85	0.08
	GNT	**2.00**	**0.19**	186.80	0.11	4.00	1.42	173.38	0.08	4.00	0.90	182.29	0.04	13.01	4.07	165.68	0.09
	GNS	5.00	1.71	170.08	0.09	**3.00**	**0.45**	179.45	0.09	5.00	1.70	187.47	0.04	12.01	4.59	159.39	0.08
20	GN	8.00	1.52	**148.35**	0.07	5.00	0.94	**154.08**	**0.06**	3.00	**1.06**	**162.87**	**0.03**	7.00	1.35	**151.41**	**0.07**
	GT	8.00	1.12	148.35	**0.07**	5.00	0.91	155.09	0.06	4.00	1.36	164.39	0.03	6.00	0.87	157.10	0.08
	GS	**4.00**	**0.96**	155.56	0.08	**3.00**	0.58	161.43	0.07	4.00	1.32	166.62	0.03	**4.00**	**0.48**	163.38	0.09
	GNN	12.00	3.19	154.93	0.08	10.00	2.26	157.55	0.06	8.00	2.86	171.49	0.03	19.01	8.22	154.22	0.08
	GNT	7.00	2.16	167.65	0.09	4.00	1.33	166.77	0.07	**2.00**	1.09	182.02	0.04	12.00	4.02	164.26	0.09
	GNS	9.00	1.87	167.87	0.09	4.00	**0.31**	178.34	0.08	4.00	1.32	189.69	0.04	15.01	6.28	159.83	0.08
100	GN	4.00	0.52	161.52	0.09	11.00	2.96	**147.84**	0.06	15.00	5.23	**157.82**	**0.03**	7.00	1.30	143.68	0.07
	GT	4.00	0.52	161.53	0.09	11.00	2.94	147.99	**0.06**	14.00	5.25	158.63	0.03	8.00	1.68	**143.66**	**0.07**
	GS	**2.00**	**0.22**	168.56	0.10	8.00	1.95	154.29	0.06	12.00	4.79	161.21	0.03	**4.00**	**0.69**	150.57	0.08
	GNN	15.00	5.41	**151.06**	**0.07**	7.00	1.56	161.76	0.07	8.00	4.19	166.02	0.03	24.01	11.96	156.11	0.08
	GNT	5.00	1.29	175.83	0.10	3.00	1.35	180.93	0.08	**2.00**	**0.48**	190.10	0.04	13.01	6.26	166.85	0.10
	GNS	6.00	1.15	167.94	0.09	**2.00**	**0.15**	183.66	0.09	3.00	1.13	191.08	0.04	12.01	7.89	159.91	0.09

GN—GARCH with normally distributed innovations (d.i.); GT—GARCH with t d.i.; GST—GARCH with skewed t d.i.; GNN—GARCHNet with normally d.i.; GNT—GARCHNet with t d.i.; GNS—GARCHNet with skewed t d.i.

Minimum cost values for each period and p pairs are in bold

Let us focus on the first two periods: starting in 2005 and 2007. Both of these periods show a high number of failures to reject the null hypothesis of the tests considered regardless of the model tested, but we can see that the GARCHNet models have better results there (the largest differences for S&P 500). The number of exceedances for GARCHNet do not show any outstanding features, but we note that the DQ test was not rejected much more often than for the standard GARCH approach. The non-linear structure of the proposed conditional variance may not be fully explained by the linear structure of the DQ test and the similar, linearly structured standard GARCH models might be outperforming NN here. In terms of statistical tests, the GARCHNet approach appears to provide models of generally higher quality. It can also be noted that the GPL statistic indicating the best predictions fell in favour of the GARCHNet models as being better in 14 out of 24 cases. The worst forecasts were made for the FTSE 100 index, where only 1 GARCHNet forecast was better. As for the GARCH(1,1) or GARCH(2,2) benchmark, it had the worst GPL in 2009, while the best in 2011.

Let us now turn to the samples starting in 2013 and 2016. In these two cases, we see a clearly higher number of rejections of the null hypotheses—both due to underestimation and overestimation of risk. In these two periods, however, the results of the GARCHNet models are in line with those of the standard GARCHs, with a slight tendency to underestimate risk (in 2016 it was mainly the GARCH models that were not rejected for the null hypothesis). On average, GARCHNet models have very similar number of exceptions. Both model families were not able to respond correctly to the COVID-19 financial market crashes, hence the high number of exceptions in the last analyzed period. It should be noted that the COVID 19 period should be seen as a stress-test for these models, and given the very similar performance of GARCHNet we would like to emphasise that it performs well under all conditions.

We note that the sequence length has a non-linear effect on the quality of the model. This depends primarily on the variability of the sample used for prediction, mainly for the standard GARCH model—see the outstanding exceptions for $p = 100$ in the sample starting in 2007 and the much numbers values for the other *p* values. This effect weakens in the case of GARCHNet, but we still notice large discrepancies for different values of *p*. Regarding the proposed length of *p*, we would suggest 20, which represents four trading weeks—one trading month and therefore gave the most remarkable results.

In Tables 4, 5 and 6 we have presented the cost function values. We note that due to the lower number of exceptions of the GARCHNet models, the cost function values of the regulator are lower than for its counterparts in most of the analyzed cases, moreover—the worst GARCHNet approaches are often better than the best GARCH in turbulent periods, while the GARCH models are better in calm periods. This is a very desirable feature of a VaR model, as in the case of an exception the potential loss is not as severe. However, from the company’s point of view, the GARCHNet models do not look so good. In most cases, the values of the company’s cost function are the worst—only in a few cases was the value of the cost function for the GARCHNet model lower. This is rather undesirable behavior due to the use of a non-linear approach. The GARCHNet model with a normal distribution appears to have the lowest ABLLF cost function value among the GARCHNet models and can usually compete with the same cost function calculated for its GARCH family counterpart. In summary, based on the cost function results, we assume that GARCHNet at this stage is a relatively conservative model. The results converge across the index tested—with noticeable differences, but these are due to the distribution of the data rather than the model specification.

## Conclusion

In this paper, we have proposed a new approach to the specification of conditional variance in GARCH models—the GARCHNet model, which incorporates a simple neural network with a long short-term memory. The idea behind the GARCHNet model is that the neural network can easily approximate non-linear relationships, and these are by far the most common in financial market volatility. Furthermore, the simplicity of the GARCH maximum likelihood estimation allows the original log likelihood functions to be used as cost functions in the GARCHNet neural network model. We proposed three different GARCHNet models, each with a different assumed distribution of innovations: normal, t and skewed t. The neural network that is used as the conditional variance specification is rather small. It contains an LSTM layer as input, followed by three fully connected layers. When the assumed distribution requires parameters other than mean and variance, these are optimized by the same neural network.

The GARCHNet models were compared with the original GARCH models in an empirical study. VAR estimates were created using a rolling window method—we trained the model using 1000 observations and estimated a forecast, then moved one time step forward and estimated another forecast. Such a procedure was repeated 250 times. Logarithmic returns of the WIG20 index (Warsaw Stock Exchange, Poland), S&P 500 (New York Stock Exchange, the USA) and FTSE 100 (London Stock Exchange, the UK) were used as data.

Our results show that GARCHNet is an outstanding model that can explain conditional variance at least at the same level as traditional approaches. Value-at-risk forecasts are rather conservative, but fewer exceptions are observed for this reason. GARCHNet would be more often chosen by regulators than by company management itself due to its relatively higher opportunity cost. We also note a rather large advantage of this model—by obtaining much more data (see *p* values greater than 10) the model can generate predictions of the same or better quality than GARCH models that consider smaller data samples.

We can see several options to enhance the model already:

1. The best lenght of sequence *p*
  *p* is one of the most influential parameters of the model, as it determines the amount of information that one forecast contains, but we did not notice any trends that would determine its impact on the quality of the model.
2. Stopping criterion
  Given that the validation sample is absent in the case of the one-day ahead forecast (time step to time step), the available options for objectively determining the end of the model training phase are exhausted. We believe that, in the case of VaR, a stopping criterion based on statistical tests would be accurate.
3. Neural network architecture and hyper-parameter tuning
  The neural network proposed here is rather small. We think that increasing the number of parameters (and thus the depth of the NN) would positively affect the quality of the model. Furthermore, we have not included any tuning of the hyperparameters—most of them have been assumed rather than tested.
4. Another approach to estimation of the distribution’s parameters
  The distribution parameters are estimated here by a separate layer that depends on the previous layers. The deteriorated performance of GARCHNet with t distribution and skewed t distribution can only be the result of the approach taken. Two other options that can be considered are either separate neural networks for the estimation of additional parameters optimized in a single procedure (parameters dependent on the forecast sample); or the inclusion of these parameters as separate weights for optimization (parameters dependent on the training sample).
5. Possible extension of this approach to time-series
  Given that GARCH models are not only used in VaR modelling, we are primarily interested in the performance of GARCHNet in traditional time series forecasting.

## Data Availability

The data that support the findings of this study are available from the corresponding author upon request.

## Code Availability

The codes that were used in this study are available from the corresponding author upon request.
